# Ancestral SARS-CoV-2-Driven Antibody Repertoire Diversity in an Unvaccinated Individual Correlates with Expanded Neutralization Breadth

**DOI:** 10.1128/spectrum.04332-22

**Published:** 2023-03-22

**Authors:** Suprit Deshpande, Mohammed Yousuf Ansari, Jyoti Sutar, Payel Das, Nitin Hingankar, Sohini Mukherjee, Priyanka Jayal, Savita Singh, Anbalagan Anantharaj, Janmejay Singh, Souvick Chattopadhyay, Sreevatsan Raghavan, Mudita Gosain, Supriya Chauhan, Shweta Shrivas, Chaman Prasad, Sangeeta Chauhan, Neha Sharma, Pradipta Jana, Ramachandran Thiruvengadam, Pallavi Kshetrapal, Nitya Wadhwa, Bhabatosh Das, Gaurav Batra, Guruprasad Medigeshi, Devin Sok, Shinjini Bhatnagar, Pramod Kumar Garg, Jayanta Bhattacharya

**Affiliations:** a IAVI-Antibody Translational Research Program, Translational Health Science & Technology Institute, Faridabad, Haryana, India; b Translational Health Science & Technology Institute, Faridabad, Haryana, India; c Bioassay Laboratory, Translational Health Science & Technology Institute, Faridabad, Haryana, India; d IAVI-Neutralizing Antibody Center and the Collaboration for AIDS Vaccine Discovery (CAVD), The Scripps Research Institute, La Jolla, California, USA; e Scripps Consortium for HIV/AIDS Vaccine Development (CHAVD), The Scripps Research Institute, La Jolla, California, USA; f IAVI, New York, New York, USA; Thomas Jefferson University

**Keywords:** Omicron, SARS-CoV-2, antibody repertoire, breakthrough infection, monoclonal antibodies, neutralizing antibodies

## Abstract

Understanding the quality of immune repertoire triggered during natural infection can provide vital clues that form the basis for development of a humoral immune response in some individuals capable of broadly neutralizing pan-SARS-CoV-2 variants. In the present study, we report variations in neutralization potential against Omicron variants of two novel neutralizing monoclonal antibodies (MAbs), THSC20.HVTR11 and THSC20.HVTR55, isolated from an unvaccinated convalescent individual that represent distinct B cell lineage origins and epitope specificity compared to five MAbs we previously reported that were isolated from the same individual. In addition, we observed neutralization of Omicron variants by plasma antibodies obtained from this particular individual postvaccination with increased magnitude. Interestingly, this observation was found to be comparable with six additional individuals who initially were also infected with ancestral SARS-CoV-2 and then received vaccines, indicating that hybrid immunity can provide robust humoral immunity likely by antibody affinity maturation. Development of a distinct antigen-specific B cell repertoire capable of producing polyclonal antibodies with distinct affinity and specificities offers the highest probability of protecting against evolving SARS-CoV-2 variants.

**IMPORTANCE** Development of robust neutralizing antibodies in SARS-CoV-2 convalescent individuals is known; however, it varies at the population level. We isolated monoclonal antibodies from an individual infected with ancestral SARS-CoV-2 in early 2020 that not only varied in their B cell lineage origin but also varied in their capability and potency to neutralize all the known variants of concern (VOCs) and currently circulating Omicron variants. This indicated establishment of unique lineages that contributed in forming a B cell repertoire in this particular individual immediately following infection, giving rise to diverse antibody responses that could complement each other in providing a broadly neutralizing polyclonal antibody response. Individuals who were able to produce polyclonal antibody responses with higher magnitude have a higher chance of being protected from evolving SARS-CoV-2 variants.

## OBSERVATION

**FIG 1:**
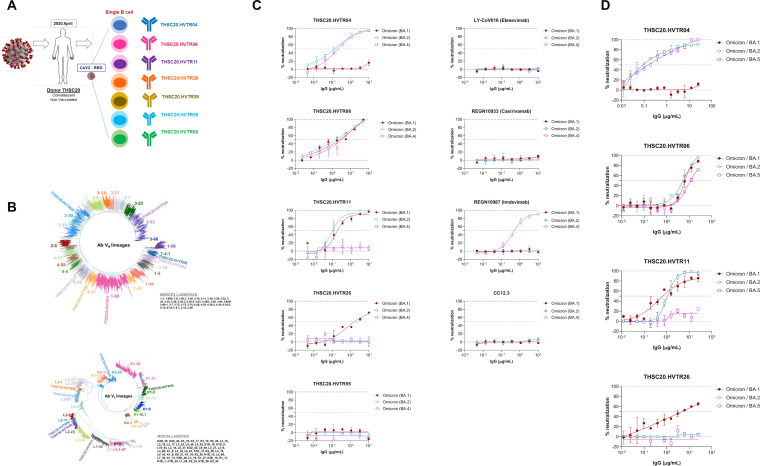
Diversity of the antibody repertoire developed in an unvaccinated individual infected with ancestral SARS-CoV-2. (A) Representation of the seven different MAbs obtained from the C-03-0020 donor infected with the ancestral SARS-CoV-2 and those originating from distinct B cell germ lines. (B) Phylogenetic clustering of maximum likelihood trees of variable heavy and light chain immunoglobulin G (IgG) genes representing the 7 neutralizing MAbs isolated from the donor C-03-0020 along with 3,441 variable heavy and light IgG chain genes representing the human B cell germ line (obtained from the CoV-AbDAb database). SARS-CoV-2 “neutralizing” monoclonal antibodies reported in the CoV-AbDAb database (http://opig.stats.ox.ac.uk/webapps/covabdab/), obtained from human germ lines, were selected based on availability of both heavy and light chain sequences (11). Postfiltration, a total of 3,448 sequences were retained (including 7 MAb sequences from the present study). Variable heavy and light chain IgG amino acid sequences were aligned using MAFFT v7.453 (13). Maximum likelihood trees were constructed with IQTree (V. 1.6.12) using a custom Ab amino acid substitution model (14, 15). The robustness of the tree was assessed using 1,000 ultrafast bootstrap replicates as well as 1,000 Shimodaira Hasegawa like approximate Likelihood Ratio Test (SH-aLRT) replicates. Trees were visualized and annotated using the ggtree package in R (16). (C) Pseudovirus neutralization assay. Neutralization of pseudoviruses expressing Omicron BA.1, BA.2, and BA.4 spikes by THSC20.HVTR04, THSC20.HVTR06, THSC20.HVTR11, and THSC20.HVTR26 MAbs was carried out using HeLa-ACE2 cells. LY-CoV016, REGN10933, REGN10987, and CC12.3 MAbs were used for comparison with our MAbs as known controls (17–20). Values are means from duplicates, and the assay was repeated at least three times. (D) Live virus neutralization assay. Neutralization potency of THSC20.HVTR04, THSC20.HVTR06, THSC20.HVTR11, and THSC20.HVTR26 MAbs against live authentic Omicron BA.1, BA.2, and BA.5 viruses was assessed in Vero E6 cells by the focus reduction neutralization test. Each point represents mean percent neutralization at a given dose of MAb plotted on the *x* axis. Neutralization curves were plotted using GraphPad Prism software (v8.1.2).

Currently, the COVID-19 pandemic is primarily driven by the Omicron lineage variants globally (https://nextstrain.org/ncov/gisaid/global/6m). Recently, a few studies highlighted a significant reduction in effectiveness of the vaccine, therapeutic monoclonal antibodies (MAbs), and natural infection-induced neutralizing antibody responses against the Omicron BA.1, BA.2, and rapidly emerging BA.4/BA.5 variants (1–6). Little is known about the antibody responses mounted in individuals previously infected with the ancestral SARS-CoV-2 (Wu-1/CoV2) prior to vaccination and capable of broadly neutralizing evolving variants of concern (VOCs), including the currently circulating and rapidly emerging Omicron variants. We previously reported isolation of five receptor binding domain (RBD)-specific neutralizing monoclonal antibodies (MAbs) from an unvaccinated Indian individual (donor C-03-0020) who was infected with ancestral SARS-CoV-2 (7). In the present study, we report the isolation and characterization of two additional MAbs from the same individual, THSC20.HVTR11 and THSC20.HVTR55 (see Fig. S1 in the supplemental material), by RBD-specific single B cell sorting and following the same pipeline reported before (7) that differed in their B cell origin. These two new MAbs varied in their ability to neutralize Omicron variants as shown in Fig. S1A. While THSC20.HVTR11 was found to neutralize Omicron BA.1 and BA.2 potently but failed to neutralize Delta (B.1.617.2) and Kappa (B.1.617.1), THSC20.HVTR55 failed to neutralize all Omicron variants (Table S1). The loss of the ability of THSC20.HVTR11 to neutralize Delta, Kappa, and Omicron BA.4/BA.5 was found to be correlated with the presence of the L452R mutation as a pseudovirus carrying an L452R substitution in the ancestral SARS-CoV-2 spike backbone conferred complete resistance to neutralization by the THSC20.HVTR11 MAb (Fig. S1B). This also indicated that leucine at the 452 position in the spike RBD (L452) is the predominant target epitope for THSC20.HVTR11 in Delta (B.1.617.2) and Kappa (B.1.617.1). Our data corroborate the ability of the L452R mutation reported to significantly abrogate the vaccine-induced antibody response and also the effectiveness of therapeutic MAbs against Delta and Omicron BA.4/BA.5 variants (5, 8–10). Both the newly isolated MAbs (THSC20.HVTR11 and THSC20.HVTR55) not only demonstrated strong binding affinity but also differed in their epitope specificities as determined by the epitope binning assay (Fig. S1C to E). Remarkably, all the seven MAbs isolated from this donor (Fig. 1A) were found to be derived from distinct B cell germ lines (IGVH3-30, IGVH7-4-1, IGVH1-69, IGVH3-53, IGVH4-39, IGVH5-51, and IGVH1-18 linked to the variable heavy chain IgG sequences and IGVL2-11, IGVL3-1, IGVL1-40, IGVL2-23, IGVL3-21, IGVK1-5, and IGVK3-20 linked to the variable light chain IgG sequences, respectively), compared with those found in the CoV-AbDAb database (11) (Fig. 1B), indicating the B cell repertoire rapidly expanded following infection, giving rise to the neutralizing antibody diversity. Interestingly, only four MAbs (THSC20.HVTR04, THSC20.HVTR06, THSC20.HVTR11, and THSC20.HVTR26) were found to variably neutralize the Omicron variants when tested as pseudoviruses (Fig. 1C) and authentic live viruses (Fig. 1D). Very interestingly, although THSC20.HVTR04 (IGVH3-30) was found to have lost its activity against BA.1 (7), presumably due to G446S and N440K mutations, it was found to potently neutralize BA.2, BA.4 (as a pseudovirus), and BA.5 (as an authentic live virus isolate) with 50% inhibitory concentration (IC_50_) values of 0.29, 0.21, and 0.19 μg/mL, respectively. On the other hand, THSC20.HVTR06 (IGVH7-4-1) could neutralize all the BA.1, BA.2, and BA.4/BA.5 variants but with low potencies, while THSC20.HVTR11 could neutralize BA.1 and BA.2 with potency comparable to that of THSC20.HVTR04; however, it failed to neutralize BA.4/BA5. The THSC20.HVTR26 MAb that we reported to moderately neutralize BA.1 (7) was included in the experiment for comparison (Table S1). The N440K mutation, which is common in Omicron BA.1, BA.2, and BA.4/BA.5 variants, did not appear to affect the neutralization potential of THSC20.HVTR04 against BA.2 and BA.4/5, though it likely had reduced potency as noted earlier with SARS-CoV-2 wild-type spike. Overall, we observed unique neutralization diversities conferred by the antibody repertoire developed in this particular individual through natural infection.

Next, we examined the magnitude and quality of neutralizing antibodies against Omicron variants developed in the same C-03-0020 donor and compared them with antibody responses observed with individuals who were vaccinated after infection with ancestral SARS-CoV-2. The participants included in this study were members of the DBT COVID-19 consortium cohort, organized by interdisciplinary research institutes and hospitals in the National Capital Region of India. The study protocol was approved by the Institute Ethics Committees of all participating institutions. Written informed consent was obtained from all the participants who contributed biospecimens and for the clinical information collected.

**FIG 2:**
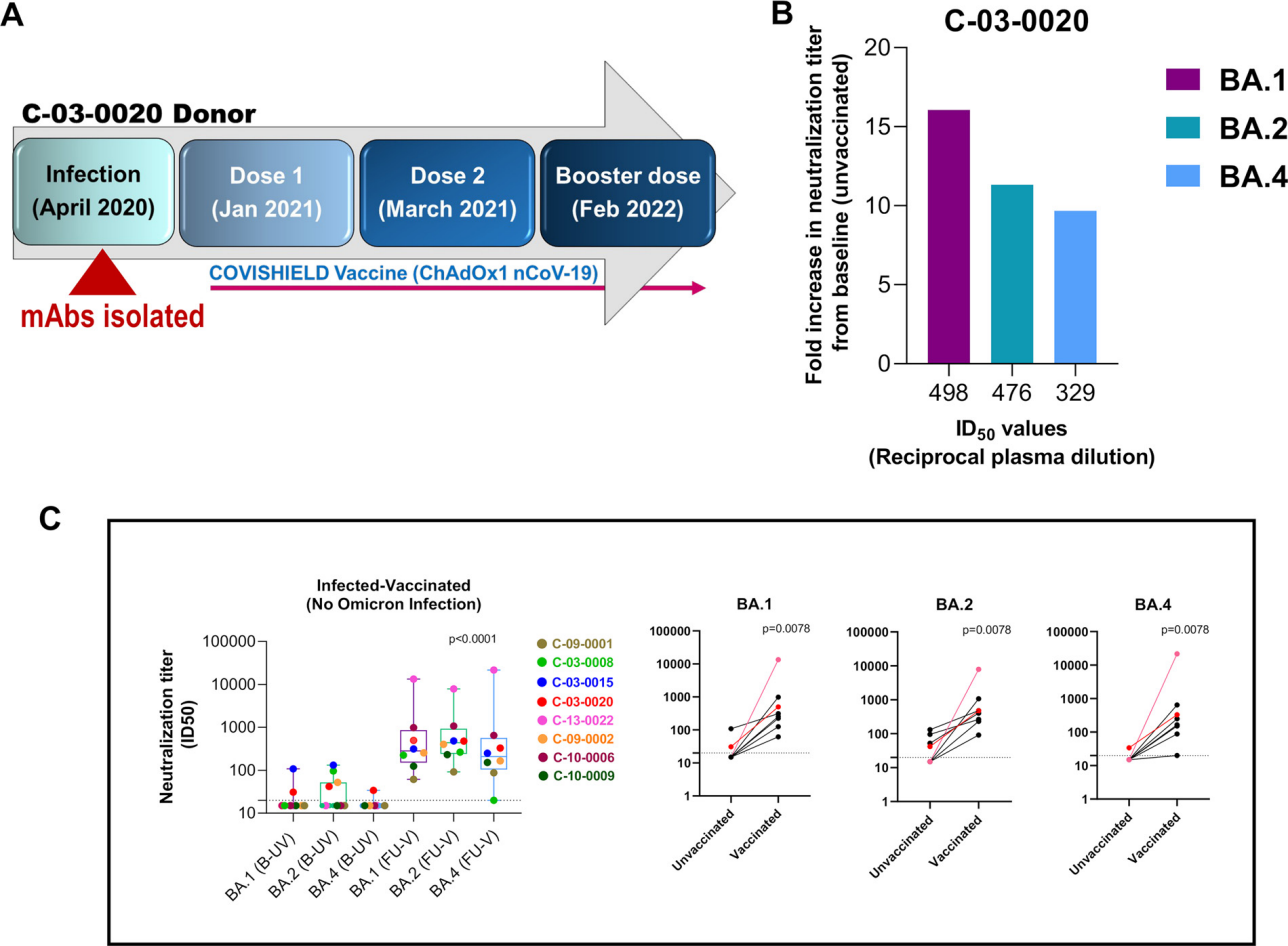
Neutralizing antibody response following vaccination in the C-03-0020 donor infected with the ancestral SARS-CoV-2. (A) Schedule of vaccine (ChAdOx1nCoV19) doses and the intervals between the doses received by the C-03-0020 donor. (B) Neutralizing antibody responses of the plasma samples obtained from the C-03-0020 donor before and after vaccination against Omicron BA.1, BA.2, and BA.4 in a pseudovirus neutralization assay. Data represent fold increase in neutralization titer postvaccination on the *y* axis and neutralization titer (50% infective dose [ID_50_]) on the *x* axis. (C) Magnitude of neutralization antibody responses (ID_50_) of plasma obtained from seven individuals infected with the ancestral SARS-CoV-2 (including the C-03-0020 donor, shown as a red dot) and their follow-up plasma samples postvaccination. The plasma sample from the C-03-0020 donor was obtained after the donor received three vaccine doses, due to the donor being lost to follow-up after receiving the second dose of the vaccine, while plasma samples were obtained from the other five donors after they received the second dose of the vaccine (see Table S2 in the supplemental material). Donor C-13-0022, infected with the ancestral SARS-CoV-2, who was included in this experiment, was not vaccinated at the time of sample collection and was used as an unvaccinated and nonbreakthrough infection control. B-UV refers to the plasma samples from baseline unvaccinated individuals, and FU-V (left panel graph) refers to follow-up vaccinated individuals postvaccination used for the neutralization assay as two groups against the pseudoviruses expressing Omicron BA.1, BA.2, and BA.4 spikes, for whom differences were found to be statistically significant (*P* < 0.001) with the Wilcoxon matched-pair signed-rank test. The right panel graph represents the same data as a head-to-head comparison of neutralization titers of the same individual before and after vaccination against individual BA.1, BA.2, and BA.4 variants. With a nonparametric Wilcoxon matched-pair signed-rank test, the *P* values were observed to be identical (*P* = 0.0078) across BA.1, BA.2, and BA.4 data sets. Neutralization curves were plotted using GraphPad Prism software (v8.1.2).

As part of this case study, we first examined the magnitude of antibodies developed in the C-03-0020 donor, 20 weeks after receiving the third vaccine dose of ChAdOx1nCoV-10 (Covishield) (Fig. 2A), for their ability to neutralize Omicron BA.1, BA.2, and BA.4. Remarkably, antibodies obtained from the follow-up visit by this individual demonstrated neutralization of Omicron BA.1, BA.2, and BA.4 with potency significantly increased by 16.06-, 11.33-, and 9.67-fold, respectively, compared to the person’s baseline convalescent antibodies (Fig. 2B). To further investigate whether what we observed with this particular individual (C-03-0020) is unique or not, we examined the ability and magnitude of antibody responses developed in six additional individuals after infection with ancestral SARS-CoV-2 and after vaccination (Table S2). Notably, we observed data from the additional six individuals comparable with those we observed with the C-03-0020 donor, who received different doses of vaccines after infection with the ancestral virus. For the C-03-0020 donor, we could obtain samples only after the person received the third vaccine dose, compared to others who received two doses of vaccines (Table S2). Additionally, we also made the following two observations: (i) antibodies elicited postvaccination from one of them (C-10-0006) neutralized all the Omicron variants (BA.1, BA.2, and BA.4) with a significantly higher magnitude (potency) and (ii) the ability of plasma antibodies obtained from other donors after vaccinations to neutralize the same Omicron variants varied—for example, antibodies elicited in donor C-03-0015 could neutralize all the Omicron variants examined in this study, while donor C-03-0008 could neutralize BA.1 and BA.2 but not BA.4 (Fig. 2C and Table S2). Although antibodies developed in these individuals before vaccination varied in neutralizing Omicron variants, the antibody response mounted postvaccination demonstrated neutralization of the same variants with increased potency (Fig. 2C), which was comparable to that observed with C-03-0020 follow-up plasma samples. Very interestingly, plasma antibodies obtained from one individual (C-13-0022) who was infected with the ancestral SARS-CoV-2 with no history of vaccination (and no history of being symptomatic, either) were found to confer neutralization of all the Omicron variants with very high potency (Fig. 2C and Table S2). This could possibly be due to development of a robust and effective SARS-CoV-2-specific B cell repertoire postinfection and possibly humoral immunity boosted by likely exposure to Omicron variants. It is to be noted that we used the plasma samples of the individual C-03-0020 obtained after the third dose of the vaccine, as opposed to others in the same category who received two doses. This was because of the unavailability of the samples after the second vaccine dose due to loss of this particular donor to follow-up.

Next, we compared the magnitude of the antibody response developed postvaccination in this particular individual (C-03-0020) with that of the response obtained from 16 vaccine breakthrough Omicron-infected individuals (Table S3). Interestingly, the plasma antibodies obtained from the C-03-0020 donor after receiving three doses of vaccine showed neutralization of the three Omicron variants examined with magnitude comparable to that observed with antibodies developed in individuals with Omicron-mediated breakthrough infection (BTI), which demonstrated the most potent neutralization of the same viruses (Fig. S2). This was observed in sharp contrast to that obtained from individuals who received full vaccine doses with no history of infection and reinfection (as shown in Fig. S2). This observation also indicates that the observed development of cross-neutralizing antibodies with higher magnitude following vaccine boosters was also likely due to clonal expansion of the B cell lineages developed in this individual through mutations within the antibody genes associated with affinity maturation as previously indicated elsewhere (12).

Overall, through this study, we made the following observations: (i) the diversity of antibody responses mounted in this particular individual (C-03-0020) immediately postinfection collectively overcame the mutational landscape offered by the evolving SARS-CoV-2 and (ii) while vaccination postinfection improved humoral immune responses, variability in their magnitude and potential to counter Omicron variants was possibly due to the differences in their ability to rapidly develop unique B cell repertoires postinfection associated with mounting of robust polyclonal antibody responses. In particular, it is likely that vaccination following infection or infection following vaccination each demonstrated hybrid humoral immunity of significantly higher magnitude. Similarly, the higher magnitude of humoral immune response due to vaccination following infection than of what we observed before vaccination in this particular C-03-0020 donor and also with other individuals in the same category (infection followed by vaccination) likely arose through affinity maturation of antigen-specific B cell lineages. Our study also provides evidence that identification of unique B cell germ lines such as IGVH3-30-3*01F, IGVH7-4-1*02F, IGVHV1-69*02F, and IGVH3-53*04F associated with development of SARS-CoV-2 variant cross-neutralizing antibodies THSC20.HVT04, THSC20.HVTR06, THSC20.HVTR11, and THSC20.HVTR26, respectively, can potentially inform rational approaches in developing improved vaccine immunogens that can selectively act to target B cell lineages in mounting humoral immune response not only of higher magnitude but also which can offer expanded breadth with increased potency against currently circulating variants, future SARS-CoV-2 variants, and potentially all coronaviruses.
