# Supplementary material for: Ancestral SARS-CoV-2-Driven Antibody Repertoire Diversity in an Unvaccinated Individual Correlates with Expanded Neutralization Breadth
Source: Microbiol Spectr. 2023 Mar 22;11(2):e04332-22. doi: 10.1128/spectrum.04332-22 (PMC10100905; doi:10.1128/spectrum.04332-22)
Supplement: Supplemental file 1 — Supplemental material. Download spectrum.04332-22-s0001.pdf, PDF file, 0.6 MB [file spectrum.04332-22-s0001.pdf]

Table S1. Neutralization breadth and potency of the mAbs isolated from infected but not vaccinated C-03-0020 donor.

SARS-CoV-2 spikes	THSC20.HVTR04[§]	THSC20.HVTR06	THSC20.HVTR11	THSC20.HVTR26[§]	THSC20.HVTR39	THSC20.HVTR55	THSC20.HVTR88
Wuhan (Wu-1)	0.005	1.22	<0.023	0.009	0.67	0.20	0.28
Alpha (B.1.1.7)	0.003	0.41	0.05	0.006	0.85	0.08	1.88
Beta (B.1.351)	0.006	1.13	0.99	0.014	1.05	0.16	0.4
Gamma (P1)	0.002	1.7	0.6	0.001	>10	0.29	0.77
Delta (B.1.617.2)	0.003	2.1	>10	0.007	1.69	0.3	0.1
Kappa (B.1.617.1)	0.008	0.91	>10	0.011	0.32	0.23	0.02
Omicron (BA.1)	>10	4.14	0.18	2.71	>10	>10	>10
Omicron (BA.2)	0.29	7.61	0.1	>10	>10	>10	>10
Omicron (BA.4)	0.21	6.58	>10	>10	>10	>10	>10

Pseudovirus neutralization assay was carried out using HeLa cells overexpressing ACE-2 receptor. Values given are doses of each mAb (in µg/mL) that conferred 50% reduction in the infectivity titer of pseudoviruses expressing different SARS-CoV-2 VOC spikes and are represented as IC_{50} values and determined by luciferase read outs in a luminometer.

§ The IC_{50} values given for THSC20.HVTR04 and THSC20.HVTR26 against SARS-CoV-2 VOCs including Omicron BA.1 variant and VOI (Kappa) were previously reported (7).

Table S2. Vaccination history of individuals previously infected with ancestral SARS-CoV-2 and neutralizing antibody responses against Omicron variants post vaccination.

PID	Infection history	Blood collection (recent)	Vaccine Doses	Vaccine name	Re-infection history	Age	Omicron Pseudovirus neutralization (ID_{50} values)		
							**BA.1**	**BA.2**	**BA.4**
C-03-0008	April 2020	20-02-2022	Two Doses	ChAdOx1 nCoV19	No	25	**225.54**	**265.31**	**<20**
C-03-0015	April 2020	27-02-2022	Two Doses	ChAdOx1 nCoV19	No	20	**313.04**	**480**	**250**
C-03-0020	April 2020	13-03-2022	Three Doses	ChAdOx1 nCoV19	No	58	**498**	**476**	**329**
C-09-0001	April 2020	17-03-2022	Two Doses	ChAdOx1 nCoV19	No	32	**61.46**	**91.5**	**87.71**
C-09-0002	April 2020	14-03-2022	Two Doses	ChAdOx1 nCoV19	No	42	**255**	**403**	**165.2**
C-10-0006	April 2020	22-03-2022	One Dose	ChAdOx1 nCoV19	No	22	**983**	**1076**	**646**
C-10-0009	April 2020	14-03-2022	Two Doses	ChAdOx1 nCoV19	No	33	**125**	**232.5**	**152**
C-13-0022[§]	April 2020	26-02-2022	None	None	No	48	**13362**	**7880.5**	**21693**

Values given are doses of each mAb that conferred 50% reduction in the infectivity titer of pseudoviruses in HeLa cells overexpressing ACE-2 receptor on surface and are represented as IC_{50} values and determined by luciferase read outs in a luminometer.

§ *Donor C-13-0022 infected with ancestral SARS-CoV-2 listed in the table was not vaccinated at the time of collection of sample (used as unvaccinated non-BTI control).*

Table S3. History of vaccination prior to infection with Omicron variant (breakthrough infection or BTI) and neutralizing antibody responses post infection against Omicron BA.1, BA.2 and BA.4 variants.

PID	Omicron variant	Blood collection	Vaccination history	Vaccine Doses	Vaccine name	Previous infection history	Age	Omicron Pseudovirus neutralization (ID_{50} values)		
								**BA.1**	**BA.2**	**BA.4**
5268	BA.1	20-02-2022	Yes	Both Doses	ChAdOx1 nCoV19	No	25	**128.71**	**188**	**36.53**
4840	BA.1	27-02-2022	Yes	Both Doses	ChAdOx1 nCoV19	No	20	**70**	**113**	**47.62**
5499	BA.1	13-03-2022	Yes	Both Doses	ChAdOx1 nCoV19	No	58	**572.61**	**964.45**	**436.74**
5507	BA.1	17-03-2022	Yes	Both Doses	ChAdOx1 nCoV19	No	32	**81.95**	**163.43**	**20**
5502	BA.1	14-03-2022	Yes	Both Doses	BBV152 Covaxin	No	42	**20**	**182.7**	**26.69**
5508	BA.1	20-03-2022	Yes	Both Doses	ChAdOx1 nCoV19	No	50	**386.37**	**315.84**	**279.1**
2058	BA.1	27-02-2022	Yes	Both Doses	BBV152 Covaxin	No	50	**315.47**	**130.27**	**20**
5512[§§]	BA.1	22-03-2022	Yes	One Dose	ChAdOx1 nCoV19	No	22	**574.31**	**411**	**316**
2076	BA.1	14-03-2022	Yes	Both Doses	ChAdOx1 nCoV19	No	33	**269.89**	**84.23**	**80.24**
5511	BA.1	22-03-2022	Yes	Both Doses	ChAdOx1 nCoV19	No	42	**547.89**	**169.74**	**20**
2071	BA.2	26-02-2022	Yes	Both Doses	BBV152 Covaxin	No	48	**274.39**	**762.47**	**288**
2077[§]	BA.2	13-03-2022	No	None	Not applicable	No	17	**20**	**23**	**20**
2176	BA.2	27-02-2022	Yes	Both Doses	BBV152 Covaxin	No	55	**286.5**	**640**	**395.41**
2079	BA.2	14-03-2022	Yes	Both Doses	ChAdOx1 nCoV19	No	28	**892**	**311.32**	**361**
5530	BA.2	06-04-2022	Yes	Both Doses	ChAdOx1 nCoV19	No	70	**31**	**616**	**20**
2080	BA.2	28-03-2022	Yes	Both Doses	ChAdOx1 nCoV19	No	31	**138**	**779**	**150**

Pseudovirus neutralization assay was carried out using plasma samples obtained from the Omicron BTI individuals 6-8 weeks post positive RT-PCR result. Values given are doses of each mAb that conferred 50% reduction in the infectivity titer of pseudoviruses in HeLa cells overexpressing ACE-2 receptor on surface and are represented as IC_{50} values and determined by luciferase read outs in a luminometer.

[§] *Donor 2077 infected with Omicron BA.2 listed in the table was not vaccinated at the time of collection of sample (used as unvaccinated non-BTI control).*

[§§] *Donor 5512 received one dose of ChAdOx1 nCoV19 vaccine at the time of collection of sample.*

A

Variable Heavy Chain IgG sequence characteristics

mAb ID	V gene & allele	% identity to germline (V region) (nucleotide)	J gene & allele	% identity to germline (J region) (nucleotide)	CDHR3 sequence	CDHR3 length
THSC20.HVTR11	IGHV1-69*02 F	97.57%	IGHJ4*02 F	100%	CARIRGYSGYGSSYYFDYW	17
THSC20.HVTR55	IGHV5-51*01 F	96.88%	IGHJ4*02 F	89.58%	CARRDRWELDPLDYW	13

Variable Light Chain IgG sequence characteristics

mAb ID	V gene & allele	% identity to germline (V region) (nucleotide)	J gene & allele	% identity to germline (J region) (nucleotide)	CDR3 sequence	CDHR3 length
THSC20.HVTR11	IGLV1-40*01F	99.65	IGLJ2*01F or IGLJ3*01F	94.74%	CQSYDSSLSHEVF	11
THSC20.HVTR55	IGKV1-5*03 F	97.85%	IGKJ2*01 F	94.87%	CQHYNGYPYTF	9

B

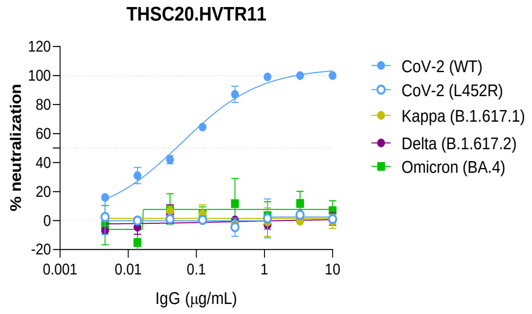

C

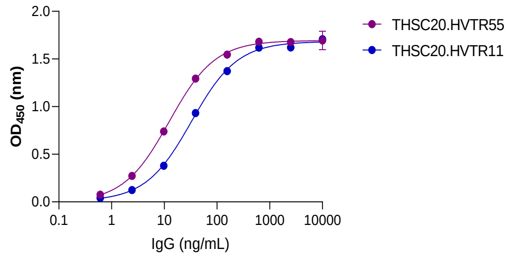

D

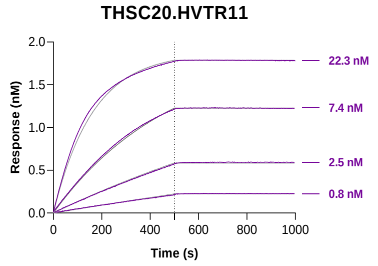

KD (M)	Kon(1/Ms)	Koff (1/s)
<1.0E-12	1.03E+05	<1.0E-07

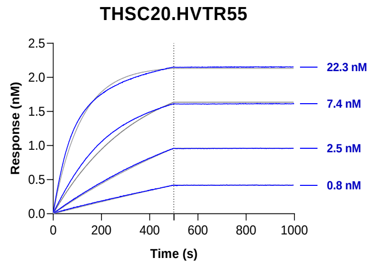

KD (M)	Kon(1/Ms)	Koff(1/s)
<1.0E-12	1.50E+05	<1.0E-07

E

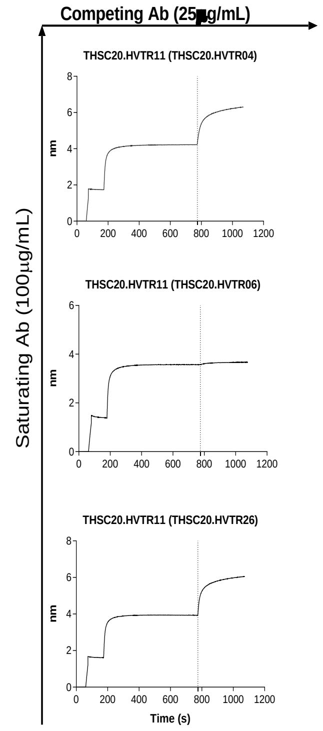

Figure S1

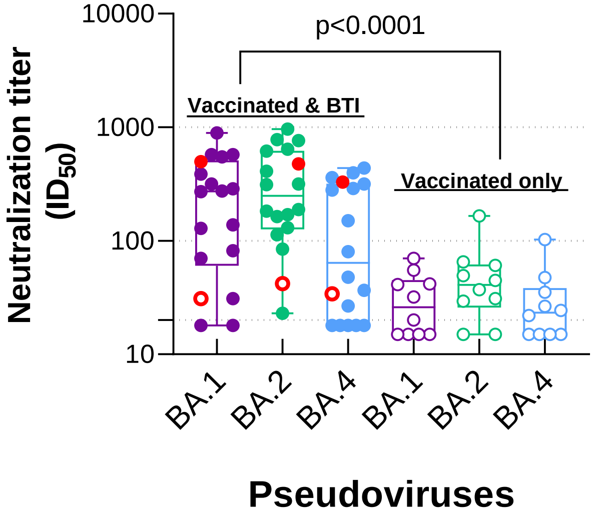
Neutralization titer
(ID50)
10000
1000
100
10
p<0.0001
Vaccinated & BTI
Vaccinated only
BA.1
BA.2
BA.4
BA.1
BA.2
BA.4
Pseudoviruses

Fig S2

Table and Figure Legends: Supplementary Information

Table S1. Neutralization breadth and potency of the mAbs isolated from infected but not vaccinated C-03-0020 donor.

Table S2. Vaccination history of individuals previously infected with ancestral SARS-CoV-2 and neutralizing antibody responses against Omicron variants post vaccination.

Table S3. History of vaccination prior to infection with Omicron variant (breakthrough infection or BTI) and neutralizing antibody responses post infection against Omicron BA.1, BA.2 and BA.4 variants.

Fig. S1. Characterization of newly isolated neutralizing mAbs. A. Nucleotide sequences of the CDRH3 and CDRL3 regions of variable heavy and light IgG chains of THSC20.HVTR11 and THSC20.HVTR55 and their B cell allelic origins as determined by using the international ImMunoGeneTics information system database (http://www.imgt.org). B. Effect of L452R substitution on the ability of THSC20.HVTR11 to neutralize SARS-CoV-2 in a pseudovirus neutralization assay. Pseudoviruses expressing spikes of Kappa (B.1.617.1), Delta (B.1.6517.2) and Omicron BA.4 which naturally contains L452R were included in the same assay. The neutralization assay was done in duplicates and was repeated at least three times. C. Binding avidity of the newly isolated THSC20.HVTR11 and THSC20.HVTR55 to ancestral SARS-CoV-2 RBD by ELISA. D. Binding affinities of THSC20.HVTR11 and THSC20.HVTR55 to the SARS-CoV-2 (Wuhan) receptor binding domain (RBD) protein by BLI-Octet. Binding kinetics of THSC20.HTR11 and THSC20.HVTR55

with SARS-CoV-2 RBD using BLI-octet analysis. His-tagged SARS-CoV-2 RBD was immobilized on NTA biosensors and binding kinetics of the IgGs to RBD was assessed using four different concentrations starting with 22.3 nM followed by 3-fold dilutions till 0.8 nM. Association and dissociation of the mAbs to the RBD bound biosensors were assessed for 500 sec each. Data shown is reference - subtracted and analyzed using Octet data analysis software v11.1 (Forte Bio Inc.). Global curve fitting using 1:1 binding model determined K_{on}, K_{off} and K_D values shown below the graphs. E. Epitope binning assay. The epitope specificity of THSC20.HVTR11 was evaluated with other three mAbs (THSC20.HVTR04, THSC20.HVTR06 and THSC20.HVTR26) which showed neutralization of Omicron variants were evaluated for epitope competition using BLI. All the incubation steps for binning experiments were performed in 1x PBS. 50-100 nM of his-tagged RBD protein antigens were loaded on Ni-NTA biosensors to achieve 0.9 to 1.3 nm of wavelength shift and then washed. Saturating concentration of mAbs (100µg/ml) was added for 10 min and competing mAbs at concentrations of 25 µg/ml were then added for 5 min in order to measure binding in the presence of saturating antibodies.

Fig S2. Comparison of the neutralization titer of antibodies developed in the C-03-0020 individual before and after vaccination with that developed in individuals with Omicron BTI and those who received two doses of vaccines (ChAdOx1nCoV19) with no history of infection. Red open and filled circles represent neutralization titer (ID50 doses) conferred by the plasma obtained from the C-03-0020 before and after receiving three doses of vaccine. ID50 refers to the reciprocal dilution of the heat-inactivated plasma samples that conferred 50% reduction in infection in a pseudovirus neutralization assay. The IC50 neutralization titers (IC50 values in µg/mL) of the four

mAbs (THSC20.HVTR04, THSC20.HVTR06, THSC20.HVTR11 and THSC20.HVTR26) that showed variable neutralization potential against the Omicron variants are plotted as reference on the right. Significant difference in neutralizing antibody response developed between Omicron BTI and vaccinated but infected individuals was observed ($p<0.0001$) using Mann-Whitney statistical test. Neutralization curves plotted using GraphPad Prism software (v8.1.2).
